# A novel image-based classification system for atlantoaxial deformity caused by mucopolysaccharidosis type IVA: an efficacy evaluation

**DOI:** 10.1186/s13018-025-06085-4

**Published:** 2025-07-15

**Authors:** Qiu-Qi Zhang, Shangguan Wen-ji, Jia Song, Zhi-Hui Liang, Fu-Chao Zhou, Hai-Tao Liu, Jiang Shao, Yue-Hui Zhang

**Affiliations:** 1 Spine Center, Xin Hua Hospital Affiliated to Shanghai Jiao Tong University School of Medicine, 1665 Kongjiang Road, Shanghai, 200092 China; 2 Baoshan Branch, Ren Ji Hospital, School of Medicine, Shanghai Jiao Tong University, Shanghai, China

**Keywords:** Mucopolysaccharidosis type IVA, Atlantoaxial deformity, Atlantoaxial dislocation, New diagnostic classification, Reliability test, Atlantoaxial fusion

## Abstract

**Study design:**

Retrospective Study.

**Objective:**

Type IVA mucopolysaccharidosis (MPS) is often associated with atlantoaxial deformity, and lacks a unified surgical treatment standard or classification system. We examined the value and clinical applicability of a new classification system for atlantoaxial deformities caused by type IVA MPS.

**Methods:**

In this single-center retrospective clinical study, we analyzed 85 patients with type IVA MPS admitted between 2018 and 2022. Using the new classification system, the patients were classified as MPS A, B, C, and D according to pathological and imaging characteristics. Intra- and interobserver consistency tests were conducted by 10 independent observers. Subsequent clinical treatment was guided by the new classification system. Demographic, surgical, and clinical data were collected.

**Results:**

Thirty-nine patients with MPS A, fifteen with MPS B, seven with MPS C, and twenty-four with MPS D were included. The inter- and intra-observer k-values were 0.773 and 0.792, respectively. During the study period, seven patients with MPS A converted to MPS D, and five underwent subsequent surgery. Two patients with MPS B converted to MPS D and underwent surgery. Two patients with MPS C underwent atlantoaxial reduction and fusion fixation, and one underwent simple posterior arch resection of the atlas. Among 33 patients who underwent posterior atlantoaxial reduction and fusion fixation, 32 achieved bone fusion. Short-term complications comprised 1 case of mortality, 1 case of postoperative airway obstruction, 1 case of pain at the iliac bone graft donor site, and 2 cases of delayed wound healing. During the long-term follow-up period, no serious surgical complications have been observed to date, and the mADI value and ASIA scores of all patients improved to varying degrees.

**Conclusion:**

The new classification system has a high reliability and clinical guidance value for diagnosis and treatment planning. The surgical plans adopted based on this diagnostic classification were effective and safe.

## Introduction

Type IVA mucopolysaccharidosis (MPS) is a common lysosomal disorder caused by mutations in the lysosomal hydrolytic enzymes. The loss of enzyme activity leads to the accumulation of intracellular glycosaminoglycans (GAGs) that affect the function of cells and impact many organs and systems throughout the body [1–3]. Extensive bone and cartilage system involvement is commonly found in patients with type IVA MPS [1, 4]. Taking the occipitocervical deformities as an example, patients with type IVA MPS often present with atlantoaxial dislocation (AAD) and/or instability and spinal stenosis. AAD originates from atlantoaxial deformities, and is primarily caused by odontoid hypoplasia. Abnormal development of the posterior arch of the atlas and the deposition of mucopolysaccharides in the anterior, odontoid, and ligamental regions of the posterior arch often result in spinal stenosis. These two pathological manifestations, either alone or in combination, may cause spinal cord compression. In severe cases, patients may experience limb weakness and numbness, unsteady gait, paralysis, other serious clinical manifestations, and sudden death [5, 6].

The treatment and classification of atlantoaxial deformities caused by type IVA MPS remain controversial. Recent advances in surgical techniques have improved surgical efficacy in these children; however, there is currently no optimal classification system for the diagnosis or treatment of this condition, including the timing of surgical intervention, due both to its rarity of the disease and the heterogeneity of pathological findings. The craniovertebral junction (CVJ) has a complex anatomical structure and close relationships with surrounding neurovascular structures. Additionally, type IVA MPS is often associated with multisystemic involvement throughout the body. Therefore, effective surgical management of CVJ deformities caused by type IVA MPS poses a significant challenge [7, 8]. Furthermore, the existing AAD classification system does not adequately integrate the pathological details of patients with type IVA MPS [9]. Therefore, we reasoned that a new classification system specific to atlantoaxial deformities caused by type IVA MPS could be established using imaging data to guide subsequent treatment plans.

## Materials and methods

### Study design

We conducted a retrospective review of children managed according to a new surgical classification system for atlantoaxial deformities caused by type IVA MPS. The study protocol was approved by the Ethics Committee of the authors’ institution, and the families of all patients provided informed consent.

### Patient population

**Fig. 1:**
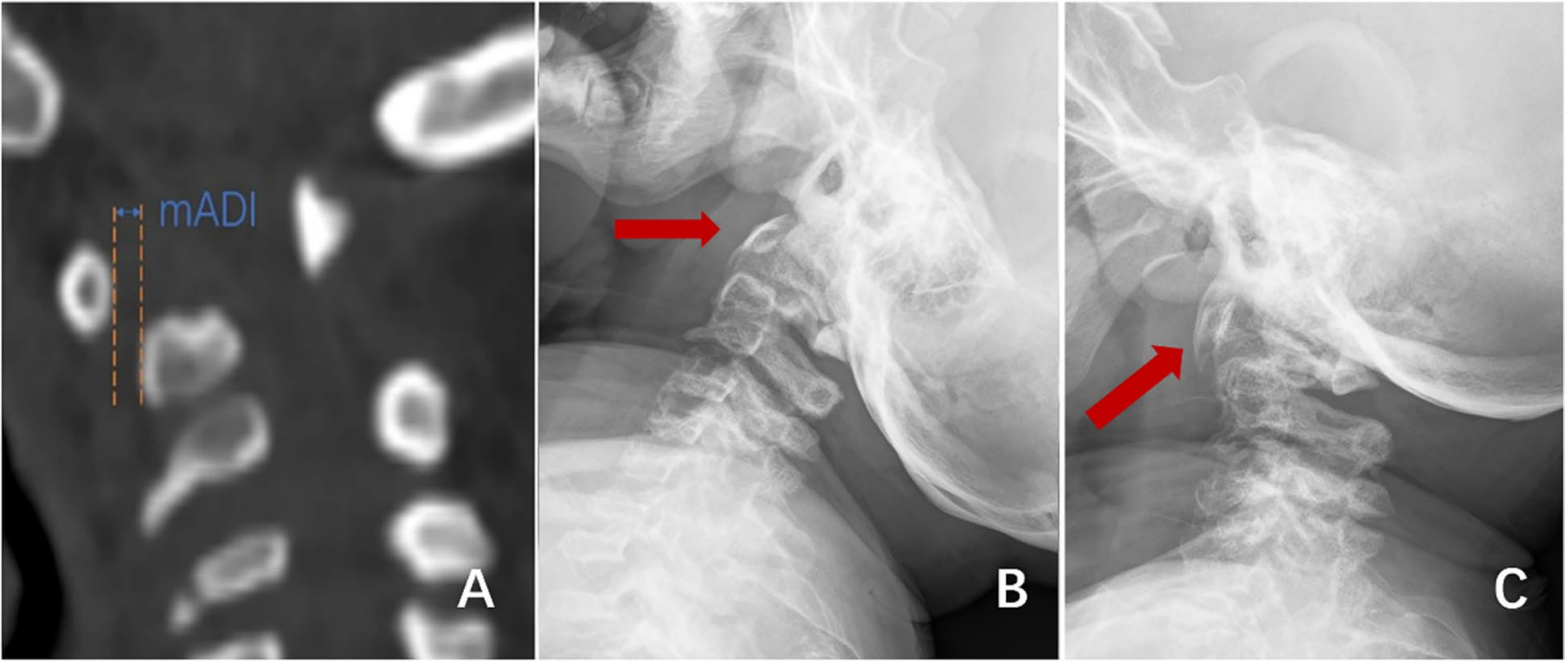
Diagram showing an overview of mADI measurement, including the distance between the posterior margin of the anterior arch of the atlas and the anterior margin of the vertebral body in the median sagittal plane of CT (**A**). Cervical dynamic radiography is used to observe cervical stability. mADI may be enlarged in the presence of atlantoaxial dislocation or instability (**B**, **C**)

Between 2018 and 2022, 112 patients with type IVA MPS were admitted to our department, of whom 85 with complete follow-up data were included in the analysis. Patients were excluded from the study if they were aged ≥ 18 years, had obvious head and neck trauma or other metabolic or neoplastic diseases, or had incomplete follow-up data and imaging information. The gold standard for MPS diagnosis is the progressive quantification of specific enzyme activities (N-acetylgalactosamine-6-sulfatase [GALNS]) in fibroblasts or leukocytes. Children with a GALNS enzyme activity of 5% lower than that of healthy people were diagnosed with type IVA MPS [10, 11]. The American Spinal Injury Association (ASIA) Impairment Scale [12] was used to evaluate neurological function and clinical outcomes. The Atlanto-Dental Interval was evaluated by measuring the distance between the anterior and posterior edges of the anterior arch of the atlas to diagnose atlantoaxial dislocation and instability. In the absence of an odontoid process, atlantoaxial dislocation was diagnosed based on a Modified Atlanto-Dental Interval (mADI) of > 5 mm (Fig. 1) [13, 14].

### Diagnostic Criteria for Spinal Stenosis

**Fig. 2:**
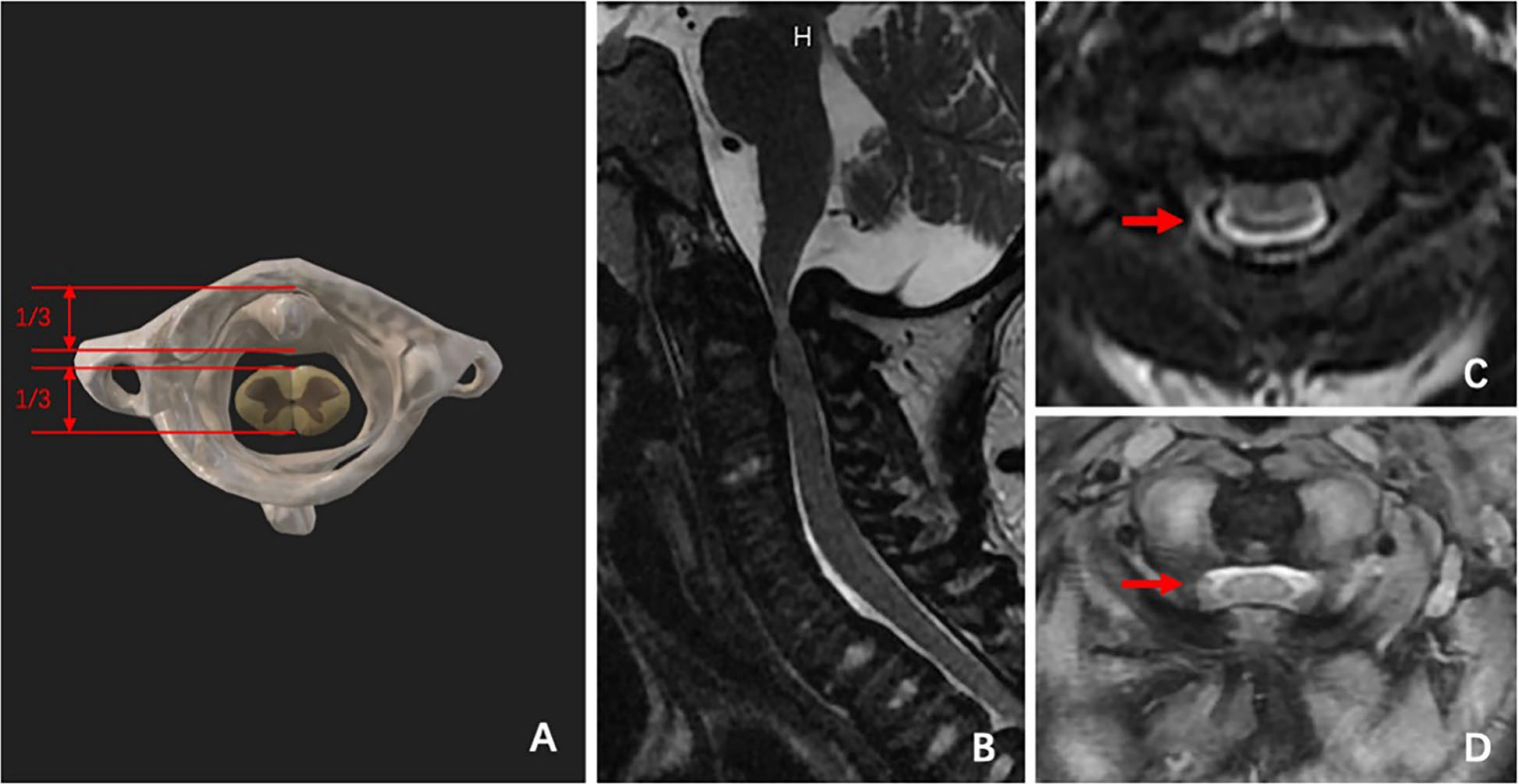
Diagnostic diagram of spinal stenosis (**A**). MRI findings of occipital cervical spinal stenosis in type IVA MPS (**B**, **C**, **D**)

Based on the data and clinical characteristics of this study group and our clinical experience, we formulated an image-based diagnostic classification for cervical spinal stenosis, which is also part of the proposed new classification. In the cross-sectional magnetic resonance imaging (MRI) view of the atlas, the odontoid process and spinal cord each occupy one-third of the sagittal diameter of the spinal canal, while the remaining one-third is taken up by the buffer space (Fig. 2A). The sagittal MRI view provides an overview of cervical stenosis and spinal cord compression (Fig. 2B). In the cross-sectional MRI view, spinal stenosis is diagnosed when ≥ 75% of the buffer space is lost at any cross-section (Fig. 2C, D). Severe spinal stenosis is indicated when > 90% of the buffer space is lost.

### Imaging

All patients underwent lateral and dynamic flexion and extension cervical radiography, computed tomography (CT), and MRI. Whole-spine radiography and MRI were performed under the guidance of an experienced surgeon to prevent severe spinal cord injury due to atlantoaxial instability during examination. Patients slated for surgery underwent CT angiography (CTA) to determine the course of the vertebral artery, echocardiography to assess cardiac function, CT-derived three-dimensional airway reconstruction, and laryngoscopy to assess airway malformations.

### New classification scheme

- MPS A: Patients diagnosed with type IVA MPS, in whom no obvious AAD or instability, significant spinal canal stenosis, or spinal cord compression was observed.
- MPS B: Patients diagnosed with with type IVA MPS in whom AAD or instability was present, but no spinal canal stenosis was observed.
- MPS C: Patients diagnosed with type IVA MPS in whom spinal canal stenosis was observed without any evidence of AAD or instability.
- MPS D: Patients diagnosed with type IVA MPS, in whom spinal canal stenosis and spinal cord compression was observed, with obvious dislocation and instability of the atlantoaxial joint.

### Reproducibility of case classification

Ten independent observers (five spinal specialists and five orthopedic fellows from four residency training hospitals in China) were selected. The average duration of the hands-on experience of the spine specialists was 14.7 years. The imaging data, medical history, and details on the condition of the patients were provided to the observers. After gaining familiarity with the new classification system and the patient information, the experts independently used these data to classify the 85 patients and subsequently completed questionnaires. For each case, the experts had to answer three questions: (1) Which subclass does this patient belong to according to the new classification system; (2) How do you propose to treat the patient? Would you select non-surgical treatment or surgical intervention?; (3) If operation is required for this patient, what surgical plan would you devise? The third question is mainly to provide guidance and reference for the formulation of treatment plan in our new classification system.

To assess the intra-observer reliability of the results, all observers were required to repeat the procedure 8 weeks after the first assessment.

### Statistical methods

Validation of the new classification system: The results were divided into intra- and interobserver reliabilities. Intra-observer reliability was measured by comparing the results of the same case observed by the same observers at different times. In contrast, interobserver reliability was measured by comparing observations of the same case by different observers during the same period. The κ-value computed via the Landis and Koch criteria was used to analyze the inter- and intra-observer consistency. The specific criteria were as follows: < 0 indicated disagreement, 0.00–0.02 indicated slight agreement, 0.21–0.40 indicated fair agreement, 0.41–0.60 indicated moderate agreement, 0.61–0.80 indicated strong agreement and 0.81–1.0 indicated almost complete agreement. Paired t-tests were performed to determine statistical differences between means. The SPSS (version 23.0; IBM, Armonk, New York, USA) software package was used for statistical analyses.

Evaluation of the clinical efficacy of the new system: Patients with MPS A were followed up every 6 months. For the other MPS types, nonsurgical patients underwent radiography and neurological function tests at 1, 2, and 4 weeks after traction initiation and at 2, 4, and 8 weeks after brace correction. Patients who underwent surgery were reviewed at 1, 3, 6, and 12 months after surgery and every 1–2 years thereafter. All patients underwent routine lateral and dynamic flexion and extension cervical radiography. The ASIA score and mADI value were measured and recorded, the improvement rate was calculated, and the changes in imaging measurements before and after surgery were recorded.

Measurement data (age, mADI, discharge time, operation time, follow-up time, etc.) were tested in accordance with normal distribution and expressed as x ± s. Enumeration data (sex, ASIA score, and imaging index) were expressed as percentages. A paired t-test was performed to compare before and after treatment, and the α value of the test level was set at 0.05.

## Results

### The image-based classification system to categorize patients with MPS

Based on the presence of AAD and instability, spinal canal stenosis, and spinal cord compression at initial presentation, 85 patients were classified with respect to diagnosis, course of disease, and clinical symptoms. This study included 39 type A (45.9%), 15 type B (17.7%), 7 type C (8.2%), and 24 type D (28.2%) patients with MPS.

### Inter- and intra-observer reliability of the new classification system

The k-values of the inter- and intra-observer reliabilities were 0.773 (strong agreement) and 0.792 (strong agreement), respectively. The inter- and intra-observer reliabilities did not differ significantly between experienced spine specialists and fellows (Table 1).

**Table 1:** Inter- and intra-observer reliability of the new classification system

	Specialist group	Fellow group	*P*-Value	K-Value
Inter-observer reliability	0.890	0.686	> 0.05	0.773
First round	0.897	0.721	-	0.801
Second round	0.884	0.650	-	0.745
Intra-observer reliability	0.901	0.730	> 0.05	0.792

### Treatment strategy

**Fig. 3:**
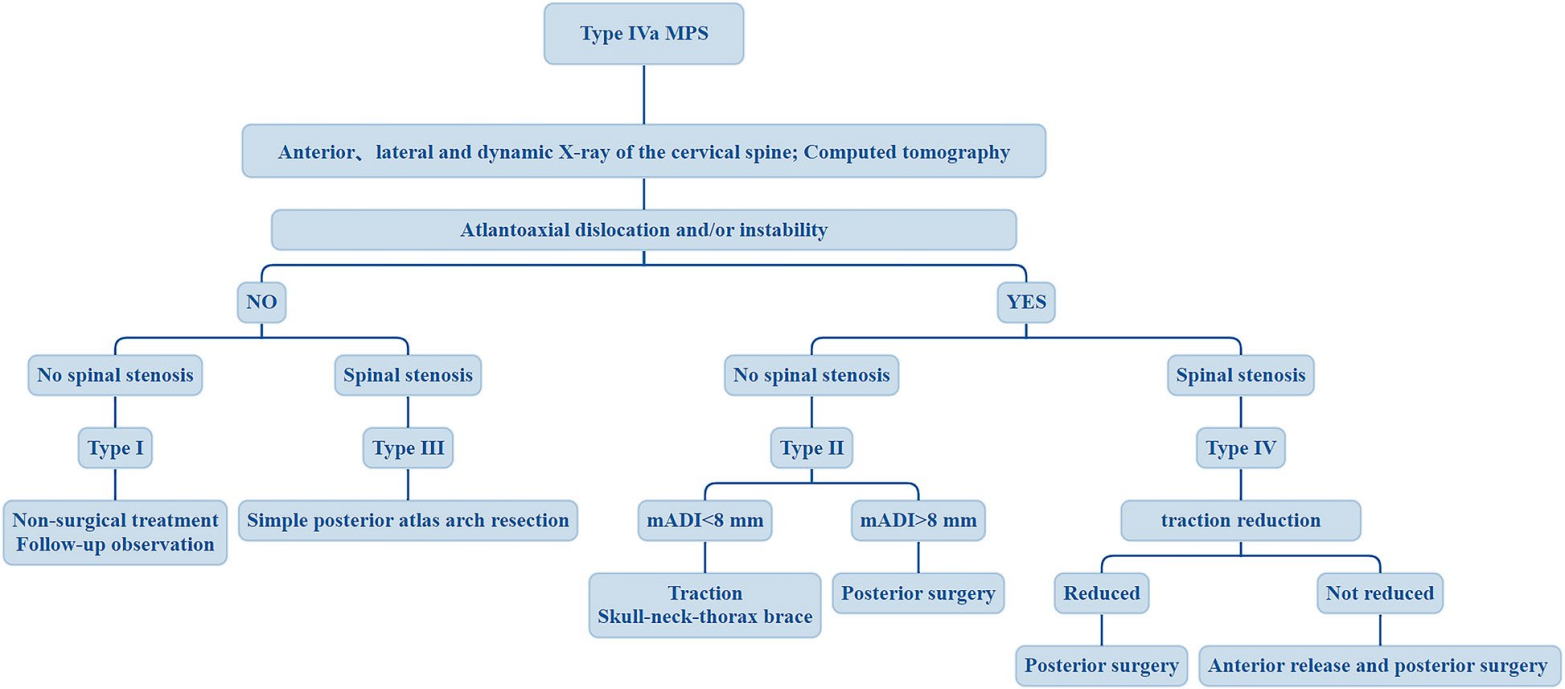
Flowchart showing the standardized surgical treatment

Treatment strategies were formulated according to the four MPS subtypes (Fig. 3). It should be noted that MPS shows a dynamic progression, and various subtypes are likely to be further aggravated, not immutable. Therefore, all patients should be closely followed throughout their life cycle to monitor the disease progression and adjust the treatment plan in time.

**Fig. 4:**
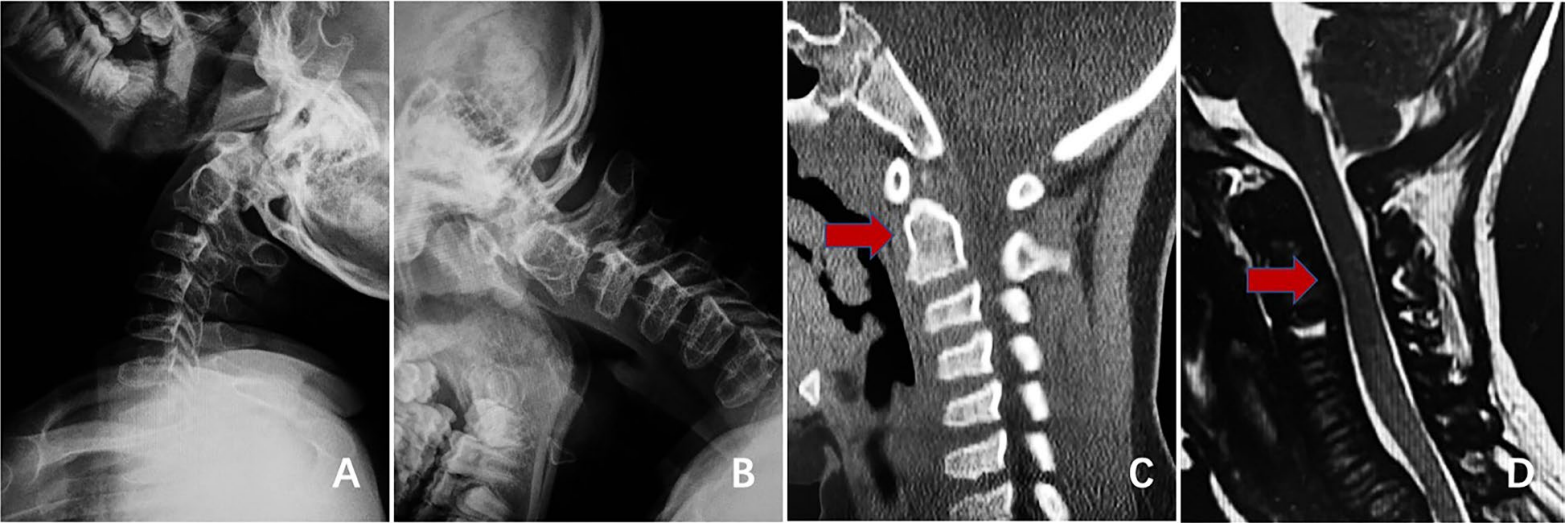
Imaging findings of a 10-year-old patient who presented to our outpatient department with a swaying gait while walking. Dynamic radiograph showing no significant atlantoaxial dislocation or instability (**A**, **B**). CT showing odontoid dysplasia. The patient is currently under follow-up observation, as there are no obvious signs of spinal stenosis or spinal cord compression (**C**, **D**)

**Fig. 5:**
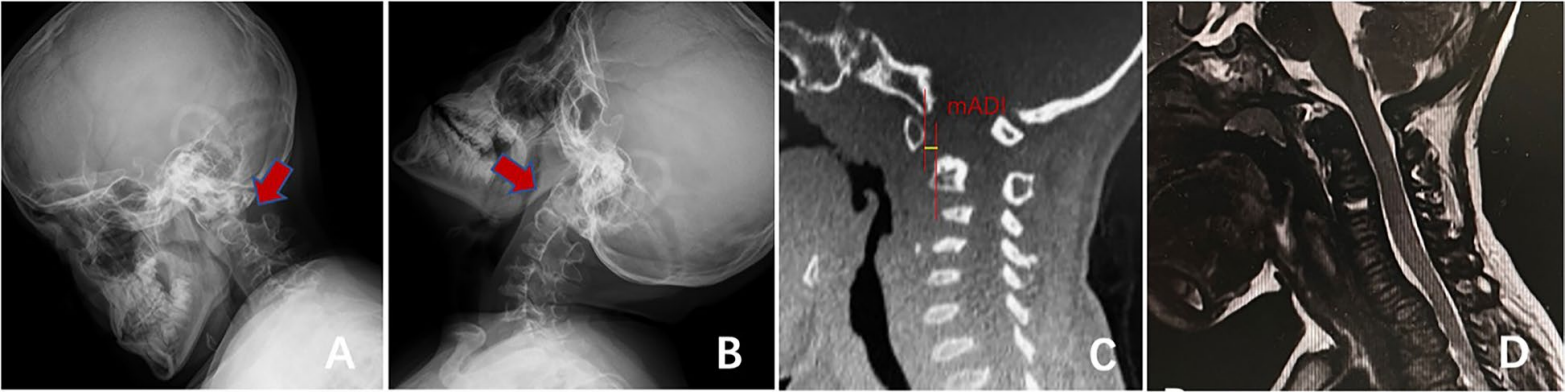
Imaging findings of an 8-year-old girl who was diagnosed with type IVA MPS. Dynamic radiograph suggesting atlantoaxial instability (**A**, **B**). CT showing the absence of odontoid process and flattened vertebrae with less smooth edges (mADI value = 5.5 mm) (**C**). MRI showing no obvious stenosis of the spinal canal, no compression of the cervical spinal cord, and no abnormal signal. Since 2021, this patient has been regularly injected with sulfatase every week and is now in the process of regular follow-up (**D**)

**Fig. 6:**
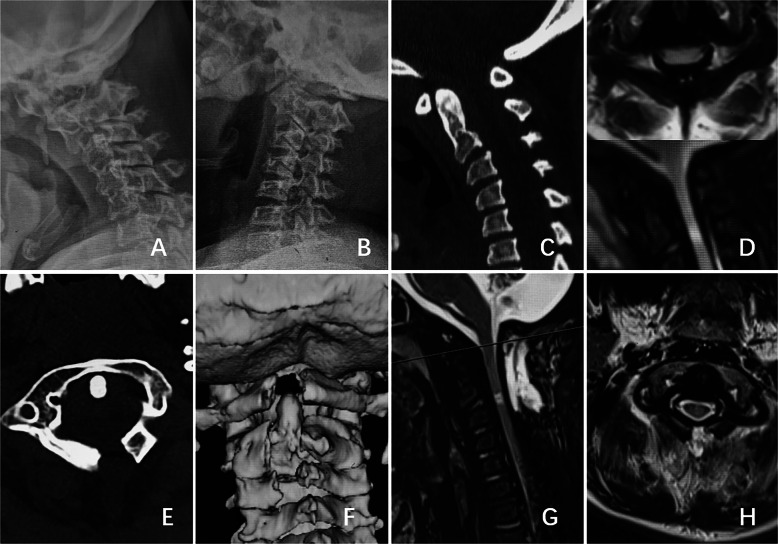
Imaging findings of a 17-year-old patient diagnosed with Type IVA MPS for more than 10 years was admitted to the hospital due to progressive decline in exercise endurance and significant restricted fine motor skills. **A, B, C** Preoperative dynamic radiograph and CT showed no significant atlantoaxial dislocation. **D:** Preoperative MRI indicating cervical spinal stenosis and spinal cord compression. **E, F** Postoperative CT showing that the posterior arch of the atlas was successfully resected. **G, H** Postoperative MRI showing that cervical spinal canal was decompress and the spinal cord compression was relieved. At the most recent follow-up, the patient reported significant improvement in symptoms, with no additional discomfort or new symptoms

- MPS A: These patients may not require surgical intervention for some time; however, close observation and regular follow-up are necessary. Tailored treatments such as genetic counseling, supportive therapies, physiotherapy, and orthopedic interventions should be offered to patients and their families (Fig. 4) [5].

- MPS B: Once AAD is confirmed, the mADI needs to be further evaluated. (1) mADI < 8 mm: Patients younger than 10 years can receive occipitomaxillary traction, and those older than 10 years can receive cranial traction or Halo frame traction. During the traction period, regular radiographic examinations should be performed to determine the reduction and close neurological function monitoring should be performed. After 4 weeks of traction, a skull-neck-thorax brace must be used for 8–10 weeks (Fig. 5). (2) mADI > 8 mm: In these patients, the atlantoaxial vertebrae are severely dislocated, structurally unstable, and prone to spinal cord compression; hence, surgery is recommended as soon as possible.

- MPS C: Such patients require further evaluation of their neurological function and atlantoaxial stability. Surgery is recommended as soon as possible in cases with significant spinal cord compression, such as spinal canal stenosis exceeding 90%, and in cases with an ASIA score above grade D. Surgery is also recommended if atlantoaxial instability worsens during follow-up. The treatment goal is to decompress the spinal canal and reduce spinal cord compression. Simple posterior atlas arch resection is recommended (Fig. 6).

- MPS D: Such patients often require surgical treatment as soon as possible to avoid serious and irreversible damage to the spinal cord.

**Fig. 7:**
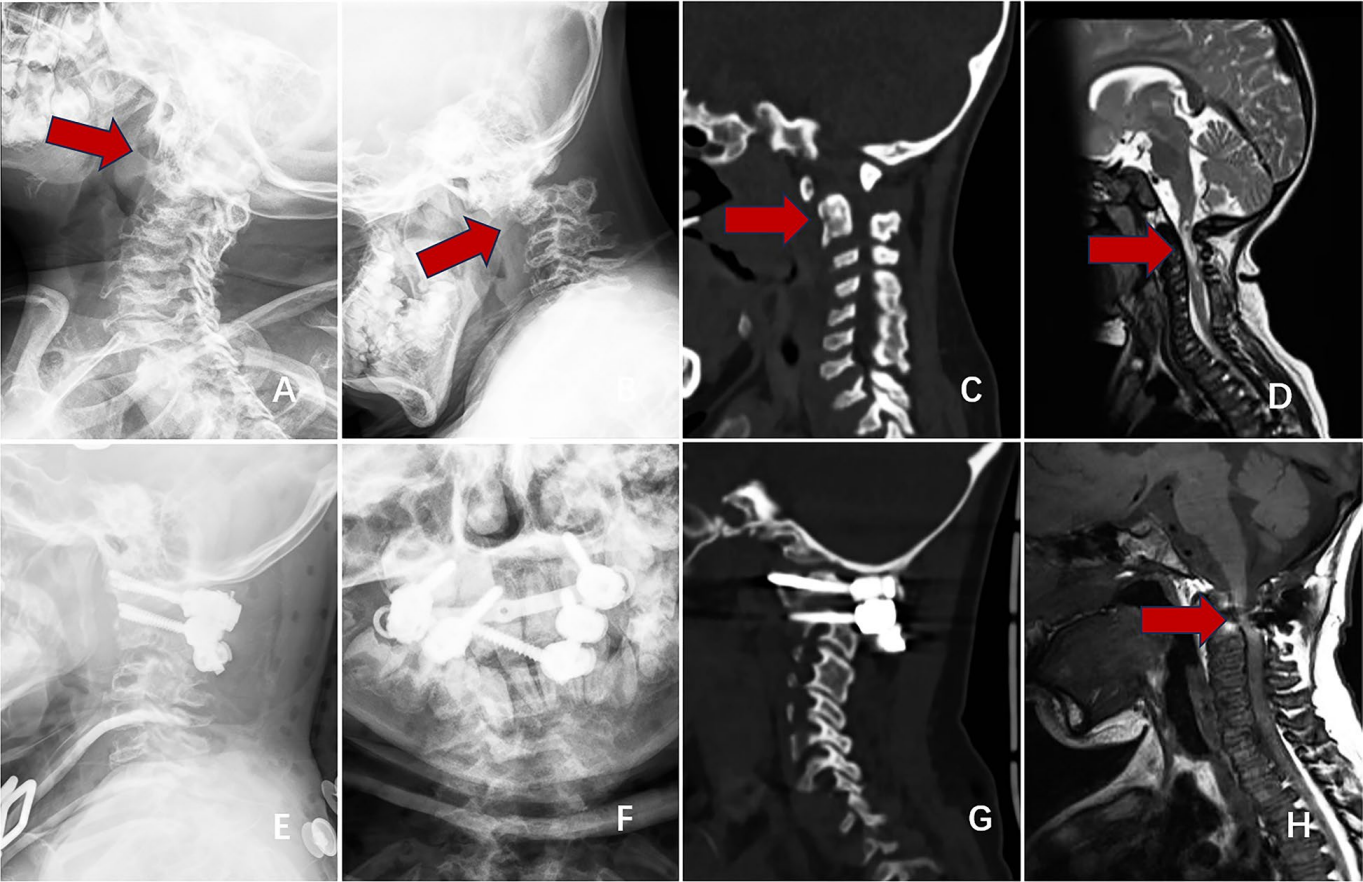
Imaging findings of an 8-year-old patient who was admitted for atlantoaxial dislocation, spinal stenosis, and spinal cord compression and subsequently underwent surgery. Preoperative dynamic radiographs indicating atlantoaxial instability and dislocation (**A**, **B**). Preoperative CT image showing the absence of the odontoid process and atlantoaxial dislocation (**C**). Preoperative MRI indicating cervical spinal stenosis and spinal cord compression (**D**). Postoperative radiography and CT showing successful atlantoaxial reduction and accurate screw insertion (**E**, **F**, **G**). Postoperative MRI showing relief of the cervical spinal cord compression (**H**)

Treatment principles include decompression, internal fixation, and fusion [15]. Posterior atlantoaxial reduction and fusion fixation via a posterior approach are recommended (Fig. 7) [16, 17].

### Evaluation of treatment modalities

None of the patients with MPS A underwent surgery at their first visit to our hospital, and all had normal neck motion and neurological function at the initial presentation. In the follow-up process, seven patients had progressive exacerbation of atlantoaxial instability, five received subsequent surgery, two were still under follow-up, and the remaining patients had no significant abnormalities.

Patients with type B are guided by traction or brace wearing according to the above criteria and received regular long-term follow-up. To this day, nine patients have normal cervical motion function and physiological curvature, while two underwent surgery; one for mADI > 8 mm and one for poor orthosis effect. Four patients are still under observation due to the short follow-up time.

Thirty-three patients received short-segment atlantoaxial reduction and fusion fixation, including five with MPS A, two with MPS B and two with MPS C who did not respond to non-surgical treatment, and one with MPS C who underwent simple posterior arch resection of the atlas. It is important to note that the specific subtypes presented here are those classified at the time of the patient's initial visit.

For patients who underwent short-segment atlantoaxial reduction and fusion fixation (Table 2), the operation time was 128.5 ± 18.1 min; intraoperative bleeding volume was 62.7 ± 20.6 ml; and duration of hospitalization was 8.0 ± 2.3 days. The duration of follow-up was 21.9 ± 8.2 months. One patient died of acute airway obstruction in the ward approximately 1 month after surgery. One patient experienced transient loss of consciousness due to airway obstruction on postoperative day 2. At the last follow-up, 32 patients had shown bone fusion in the bone graft area. The mADI value was 4–12 mm before surgery (average 7.2 ± 2.7 mm) and 0–4 mm after surgery (average 1.8 ± 1.0 mm; *P* < 0.05). At the last follow-up, the ASIA scores of all patients had improved to varying degrees. All differences were considered statistically significant. The stabiliy and normal physiological sequence of the cervical spine were reconstructed, and the activity function and physiological curvature were within the normal range.

**Table 2:** Demographic and clinical data of the 33 patients who underwent short-segment atlantoaxial reduction and fusion fixation via the posterior approach

No	Sex/Age (years)	mAD I(mm)		ASIA score		Length of stay (days)	Operation duration (minutes)	Blood loss (milliliter)	FU (months)
		Pre-	Final-FU	Pre-	Final-FU				
MPS I									
1	F/7.5	6	2	d	e	7	125	50	29
2	F/9	8	2	e	e	8	130	55	33
3	M/12	5	1	d	e	8	130	65	28
4	M/11	7	2	d	e	11	110	70	31
5	F/7	5	1	e	e	6	125	50	30
MPS II									
6	M/9	7	2	e	e	11	130	70	32
7	M/11	9	2	d	e	12	115	55	27
MPSIII									
8	M/5	5	1	d	d	7	125	45	13
9	F/13	4	2	c	d	8	125	50	21
MPSIV IV									
10	M/8.0	12	4	c	d	7	110	65	36
11	M/4.2	10	3	c	d	11	160	105	34
12	F/9.0	5	4	d	e	7	115	60	33
13	M/9.0	6	4	d	e	12	165	100	33
14	F/2	9	3	c	d	9	155	70	25
15	M/3	5	1	d	e	7	115	70	23
16	M/9	6	0	e	e	5	95	20	23
17	M/5	6	1	d	e	9	140	90	22
18	F/9	7	2	d	e	6	135	65	21
19	M/1.9	6	0	d	e	10	130	95	19
20	F/1.3	7	1	d	e	7	120	60	18
21	F/13	9	1	d	d	10	150	85	17
22	F/15	17	2	c	Death	14	170	80	Death
23	M/6	6	2	d	e	7	140	70	16
24	M/8	5	1	d	d	10	120	70	16
25	M/6	6	2	d	e	6	115	60	15
26	F/6	7	2	d	e	6	115	70	14
27	M/15	14	3	c	d	9	150	75	14
28	M/6	6	2	d	e	6	120	80	13
29	M/7	6	1	e	e	5	100	25	22
30	M/9	6	2	e	e	5	100	25	19
31	F6	7	2	d	e	6	135	50	27
32	F/7	8	2	d	e	6	130	40	21
33	F/11	6	1	e	e	6	140	30	26
		*P* <.05		*P* <.05					

Thirty-three patients received short-segment atlantoaxial reduction and fusion fixation via a posterior approach including five with MPS I and two with MPS II who did not respond to conservative treatment

*F* female, *M* male, *pre-* pre-operation, *FU* follow-up, *mADI* modified atlantodental interval, *ASIA* American Spinal Injury Association scale

## Discussion

Type IVA MPS was first described in four Swedish siblings in 1929 by Morquio et al., and was systematically described by Brailsford et al. in the UK in the same year [18, 19]. Type IVA MPS is an autosomal recessive disease that affects all systems of the body, but particularly the skeletal system. Patients often have no obvious abnormal symptoms at birth, but gradually develop severe skeletal abnormalities with age. Spinal deformity or dysplasia predominantly occurs in the occipitocervical and thoracolumbar segments. Occipitocervical lesions often lead to cervical spinal cord compression and subsequent clinical manifestations, including unsteady gait, limb weakness, numbness, activity disorders, and even paralysis, that can seriously threaten normal physiological activities and life in children [20, 21].

Currently, there is no consensus on the surgical diagnosis and treatment plan for occipital and neck lesions in patients with type IVA MPS, and long-term and systematic clinical research on this topic is lacking. First, owing to the pathological manifestations, the classification systems we previously followed, such as the Stauffer and TOI classification systems [22], are not fully applicable to type IVA MPS, likely leading to excessive or inappropriate medical treatment. Second, it is unclear whether surgery is the best plan for all such patients, or what clinical interventions should be performed in patients with definite occipito-neck skeletal malformations according to different clinical manifestations. Our new classification system provides solutions to the aforementioned problems.

Although most patients with type IVA MPS have occipitocervical deformities, we recommend regular follow-up and non-surgical treatment as the first choice for patients without significant spinal cord or nerve compression, AAD, or instability. With the application of current medical treatments such as enzyme replacement therapy and hematopoietic stem cell transplantation, disease progression can be delayed to a certain extent. For patients with mild AAD and instability alone, standardized and rigorous traction is necessary, accompanied by close follow-up and monitoring of neurological function. Early surgical treatment is indicated when imaging examinations suggest spinal stenosis and spinal cord compression, especially when further neurological function assessment suggests decreased muscle strength, increased muscle tone, and positive pathological signs [23].

The new classification system we propose has several advantages. First, we explored and defined the diagnostic criteria for atlanto-occipital spinal stenosis. We have observed that most patients with type IVA MPS have varying degrees of spinal stenosis. However, the existing classification of occipitocervical deformities often does not include stenosis. In our definition, spinal stenosis caused by AAD and other factors was excluded, and we primarily focused on the typical pathological manifestations of type IVA MPS. This included atlas dysplasia, such as posterior atlas arch non-union and indentation, as well as mucopolysaccharide hyperplasia and accumulation in the atlantoaxial region. Second, through the analysis of spinal stenosis, the new classification provides a good guide for subsequent surgical treatment. Once a patient is diagnosed with MPS C, the neurological function or degree of spinal stenosis should be evaluated further. When the ASIA score is above grade D, or severe spinal stenosis is present (≥ 90% of the buffer space is lost), surgical treatment should be performed as soon as possible. Although patients with MPS C are temporarily free of AAD and instability, the atlantoaxial region still lacks stability and firmness due to the absence of the odontoid process. In summary, > 70% of patients with MPS C tend to require surgery. Posterior arch resection of the atlas is simple and effective and is recommended as early as possible before the onset of clinical symptoms and neurological damage; however, due to atlantoaxial dysplasia in these patients, there is still a potential for instability and dislocation in the later stage; hence, reduction and fusion fixation surgery may be the best option for these patients. Conversely, immediate surgery is often recommended for all patients with MPS D because of their unstable condition. The critical aspect of the surgical treatment of these children is to relieve spinal cord compression, correct AAD and instability, and reconstruct the normal sequence and stability of the atlantoaxial vertebrae to avoid irreversible damage to nerves and spinal function [24, 25].

Traditional surgical approaches for atlantoaxial dislocation include anterior surgery, posterior surgery, and combined anterior and posterior surgery. Over the past decade, advances in surgical techniques and technologies have introduced new methods for reduction and decompression, such as posterior intra-articular release of the atlantoaxial facet joint, transnasal odontoidectomy, and osteotomy of the fused atlantoaxial facet joint using an ultrasonic bone scalpel [7, 26]. Given the unique characteristics of these children, in this study, we recommend posterior atlantoaxial reduction and fusion fixation for patients with MPS D, and the postoperative atlantoaxial instability and dislocation in the children were resolved satisfactorily. Neurological function improved significantly during follow-up. An iliac bone autograft was used to achieve bone fusion in the shortest possible time. Follow-up results 6 months after surgery showed that all patients achieved bone fusion, further confirming the reliability of iliac autogenous bone selection. One patient with adenoid hypertrophy and airway stenosis underwent tracheotomy approximately 1 week after surgery due to difficulty in extubation, and eventually died of fatal airway obstruction 1 month after surgery. Another patient experienced airway obstruction and transient loss of consciousness 1 day after surgery. Other surgical complications included iliac pain in one patient and delayed wound healing in two patients. Since these patients often have complications of systemic multi-organ and multisystem lesions, perioperative management should be further improved in the future [27].

Our new classification of type IVA MPS followed the standardized process of creating new classification systems, and the research mentioned above showed that our new system was highly reliable based on both inter- and intra-observer parameters. The inter- and intra-observer k-values for the reliability and choice of treatment were 0.773 and 0.792, respectively. No clear difference was observed between specialists and fellows in this respect. Our results indicated that this new classification system is highly reliable and can be used safely in clinical practice.

This study has several limitations which should be mentioned. First, due to the rarity of type IVA MPS, the number of cases included was small, and no control group of patients was included. Therefore, future studies with larger sample sizes should be conducted. Second, the sensitivity and specificity of the new system could not be confirmed at this time because even experienced spinal specialists may dispute the diagnosis of specific types of IVA MPS and the choice of treatment options indicated. External validation of the diagnosis will be included in our subsequent study to further confirm the feasibility of this classification. Lastly, longer-term follow-up is warranted to determine surgical outcomes and any neurological changes.

## Conclusion

The new imaging-based MPS diagnosis and classification system presented herein is clear in concept and definition, and the logical relationship between our classification and clinical treatment is rigorous and robust.

## Data Availability

No datasets were generated or analysed during the current study.
